# Supplementary material for: Target enrichment improves culture-independent detection of Neisseria gonorrhoeae and antimicrobial resistance determinants direct from clinical samples with Nanopore sequencing
Source: Microb Genom. 2024 Mar 26;10(3):001208. doi: 10.1099/mgen.0.001208 (PMC10995632; doi:10.1099/mgen.0.001208)
Supplement: Uncited Supplementary Material 1. [file mgen-10-01208-s001.pdf]

Supplementary Materials

Table S1. *Neisseria gonorrhoeae* genomes used for SureSelect custom probe library design. WHO reference genomes were included as these have been extensively characterised and represent the major known resistance mechanisms between them. The addition of H18-209 was to include a recent representative of the FC428 clone given concern about the spread of this ceftriaxone resistant lineage. NCCP11945 was included as this is a frequently used reference genome in whole-genome sequencing studies.

***N. gonorrhoeae* strain**	**BioSample number**	**BioProject number**
H18-209/ SRX5491102 (1)	SAMN11081007	PRJNA523794
NCCP11945	SAMN02603475	PRJNA29335
WHO F	SAMEA2448460	PRJEB14020
WHO G	SAMEA2448461	PRJEB14020
WHO K	SAMEA2448462	PRJEB14020
WHO L	SAMEA2448463	PRJEB14020
WHO M	SAMEA2448464	PRJEB14020
WHO N	SAMEA2448465	PRJEB14020
WHO O	SAMEA2448466	PRJEB14020
WHO P	SAMEA2448467	PRJEB14020
WHO Q	SAMEA4640835	PRJEB26560
WHO U	SAMEA2796327	PRJEB14020
WHO V	SAMEA2796328	PRJEB14020
WHO W	SAMEA2448470	PRJEB14020
WHO X	SAMEA2448468	PRJEB14020
WHO Y	SAMEA2448469	PRJEB14020
WHO Z	SAMEA2796326	PRJEB14020

Table S2. Genes involved in conferring antimicrobial resistance, represented 10-fold in the final SureSelect custom probe library.

**Gene**	**Antimicrobial agents**
23S rRNA	Macrolides
gyrA	Fluroquinolones
ermB	Macrolides
ermC	Macrolides
ermF	Macrolides
mef	Macrolides
mtrR	Macrolides, cephalosporins, penicillin, tetracycline
parC	Fluroquinolones
penA	Cephalosporins, penicillin
ponA	Cephalosporins, penicillin
porB	Cephalosporins, penicillin, tetracycline
blaTEM-1	Penicillin
blaTEM-135	Penicillin
tetM	Tetracycline

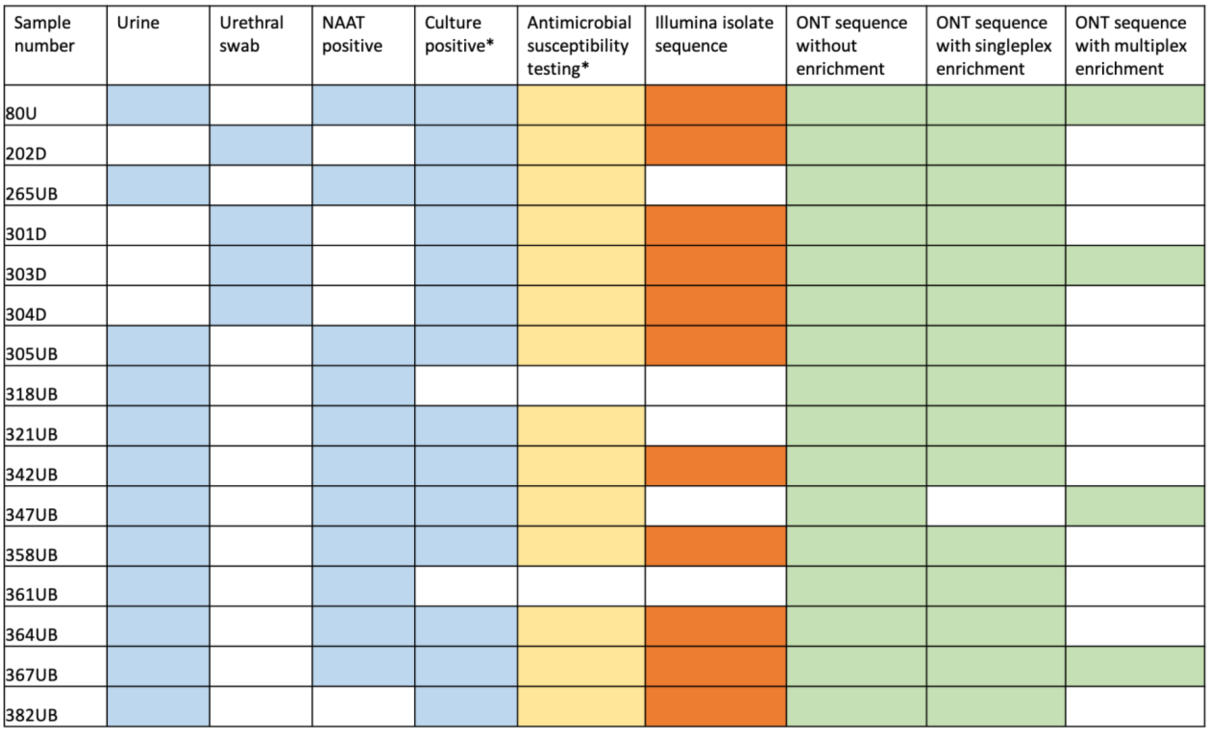

Figure S1. Sample information (blue) and summary of data available for each individual sample: Antimicrobial susceptibility data (yellow); Illumina sequence data from isolate culture (orange); ONT sequence data from urine or urethral swab extract (green). *culture-positive status and antimicrobial susceptibility results reported for urine samples were derived from culture of a corresponding swab collected at the same time as the urine sample.

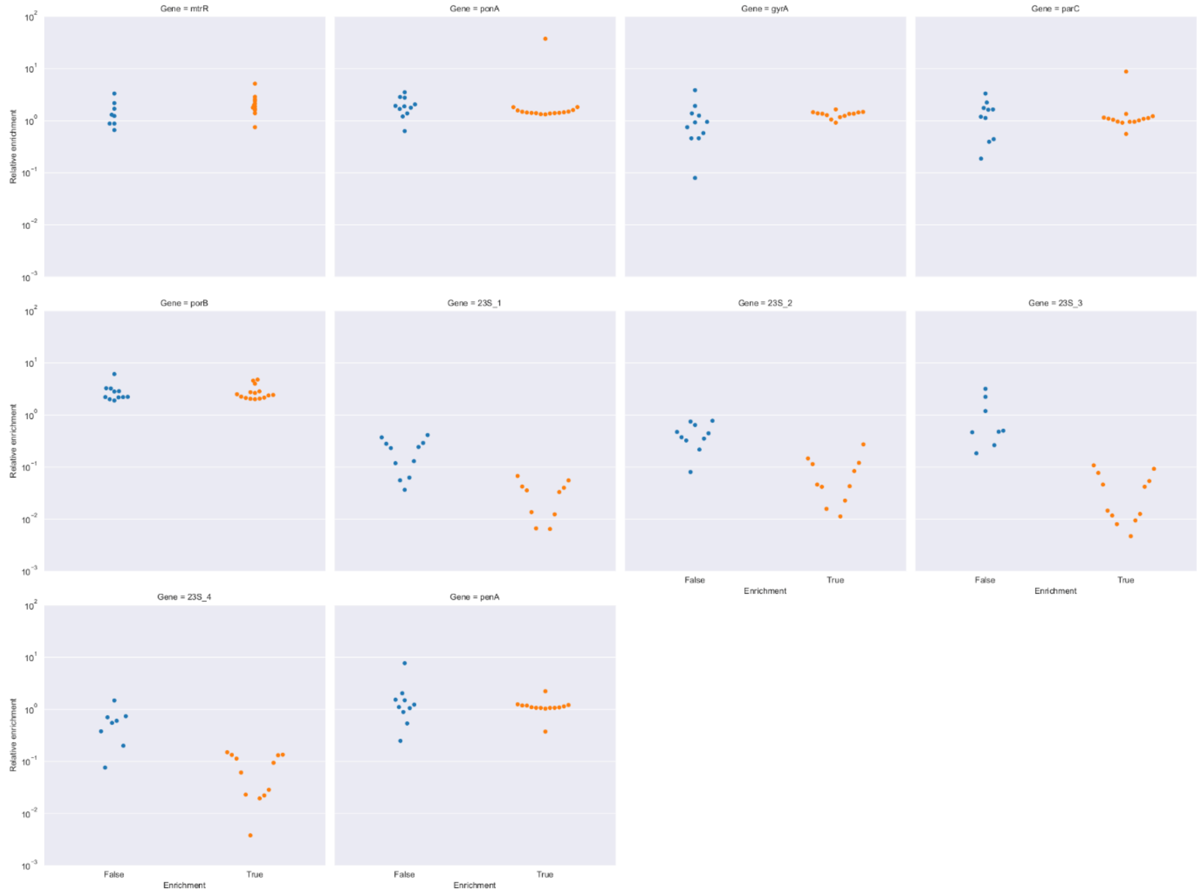

Figure S2. Relative enrichment of AMR genes in comparison to whole genome. Blue represents mean depth of AMR genes relative to mean genome depth before enrichment; orange represents mean depth relative to mean genome depth after singleplex enrichment for 23S, gyrA, mtrR, parC, penA, ponA, porB

References

1. Eyre DW, Town K, Street T, Barker L, Sanderson N, Cole MJ, et al. Detection in the United Kingdom of the Neisseria gonorrhoeae {FC428 clone, with ceftriaxone resistance and intermediate resistance to azithromycin, October to December 2018. Euro Surveill. 2019 Mar;24(10).
